# Supplementary material for: Zn (II) Porphyrin Built-in D–A Covalent Organic Framework for Efficient Photocatalytic H2 Evolution
Source: Polymers (Basel). 2022 Nov 13;14(22):4893. doi: 10.3390/polym14224893 (PMC9696642; doi:10.3390/polym14224893)
Supplement: Supplementary file 1 [file polymers-14-04893-s001.zip › polymers-2007857-supplementary.pdf]

Supplementary Information for

Zn (II) porphyrin built-in D-A covalent organic framework for efficient photocatalytic H_2 evolution

Mingbo Lv, Xitong Ren, Ronghui Cao, Zhiming Chang, Xiao Chang, Feng Bai* and Yusen Li*

Section S1. Materials and Methods

1.1. Materials

5,10,15,20-Tetrakis-(4-aminophenyl)-porphyrin and 5,10,15,20-Tetrakis-(4-aminophenyl)-porphyrin-Zn-(II) was synthesized by the previously reported method[1], thieno[3,2-b]thiophene-2,5-dicarboxaldehyde (TT) was purchased from Wohler Organic, ascorbic acid (AA), N,N-dimethylformamide (DMF), 1,4-phthalaldehyde pyrrole and sodium sulphate (Na_2SO_4) was purchased from Aladdin, ethanol, 2-hydroxypropanoic acid, tetrahydrofuran and acetone was purchased from FuYu Reagent, 4-nitrobenzaldehyde and 1,4-phthalaldehyde was purchased from Mackin, hexadecyl trimethyl ammonium bromide (CTAB) and potassium hexachloroplatinate (K_2PtCl_6) was purchase from J&K Scientific. All purchased reagents were immediately used without any further purification.

1.2. General methods

^{1}H nuclear magnetic resonance (NMR) spectra were tested on a Bruker AVANCE III HD500 (Bruker, Germany). Solid-state ^{13}C CP/MAS NMR spectra were recorded on a Bruker 400 (Bruker, Germany) at the resonance frequency of 100.62MHz. Powder X-ray diffraction (PXRD) were recorded on a bruker D8-ADVANCE (Bruker, Germany). Scanning electron microscopy (SEM) and Transmission electron microscope (TEM) used separately Nova Nano SEM 450 (FEI, American) and JEM-2100&JEOL JEM-F200 (JEOL, Japan). X-ray photoelectron spectroscopy (XPS) measurements were measured on an AXIS SUPRA+ (KRATOS, Japan). Fourier transform infrared (FT-IR) spectra were performed on a Bruker VERTEX 70 (Bruker, Germany). Ultraviolet-visible diffuse reflectance spectra (UV-Vis DRS) were recorded on a Cary 5000 (Agilent, American) at room temperature. Nitrogen physisorption analyses were evaluated by an Autosorb IQ (Quantachrome, USA) at 77 K. Thermogravimetric analyses (TGA)were recorded on a TGA/DSC3+ (METTLER TOLEDO, Switzerland).

1.3 Photocatalytic measurements

The photocatalytic experiments were carried out on the photocatalytic system of CEL-SPH2N (CEAULIGHT, China) with the light power provided by a 300 W Xenon lamp (CEL-HXF300/CEL-HXUV300, China). The Mott-Schottky plots and photocurrent response were conducted on an electrochemical workstation (Autolab, Holland) in a standard three-electrode system. The as-prepared samples coated on FTO substrate, Ag/AgCl electrode and a platinum plate were used as working, reference and counter electrodes, respectively. The experiments were performed in the 0.1 M Na_2SO4 electrolyte solution purged with N_2 to remove O_2 before any measurements. The photocurrent density was recorded under visible light switching on and off mode ($\lambda > 400$ nm, 300 W Xenon lamp). The production of hydrogen was analyzed by gas chromatography (GC7920) with nitrogen as its carrier gas. The hydrogen generator (HGH-300E) of Beijing Huilong Company was used to obtain high-purity hydrogen and then the corresponding hydrogen standard curve as well as the standard curve equation (R^2=0.9999) were made.

The specific experimental process is as follows. Adding COF powder (5 mg) and AA (0.88

g) into 50 ml of water, fully vibrate in ultrasonic device until the COF material is completely dispersed and AA is completely dissolved, and then 1.3 ml (5 wt %) of 1 mM K_2PtCl_6 is added into the solution and the mixture is sonicated for another 3 minutes, adjusting the pH by titration with 2 M NaOH solution. After completing the above steps, 30 min air extraction was needed before the analysis was started, in order to ensure the complete extraction of air, what's more, the temperature of the system was controlled at 4 °C, and a 400 ± 20 nm filter was used.

Section S2. Synthetic Procedures

Synthesis of 5,10,15,20-Tetrakis (4-Nitrophenyl) porphyrin (TNPP) [S2]

A solution of nitrobenzene (25 mL) and lactic acid (7 mL) were heated up to refluxing, then distilled pyrrole (1.84 g, 27.5 mmol) and 4-nitrobenzaldehyde (4.00 g, 27.5 mmol) dissolved in nitrobenzene (12 mL) were slowly added into the reaction flask in 20 min. The mixture was stirred at 135 °C for 2 h. After cooling to 60 °C, methyl alcohol (15 mL) was injected, the crude product was collected by filtration and washed with methyl alcohol for three times. The product was dried at 60 °C in vacuum to gained crystalline purple powder TNPP (1.2 g, 22%).

5,10,15,20-Tetrakis-(4-aminophenyl)-porphyrin [1]

At room temperature, TNPP (1.65 g, 2.05 mmol) and concentrated hydrochloric acid (75 mL) were added into a 250 mL round-bottom flask, then $SnCl_2{\cdot}2H_2O$ (7.00 g, 29 mmol) was dissolved in concentrated hydrochloric acid (20 mL) and dropwise added in 10 min. The mixture was stirred at room temperature for 2.5 h, following warmed up to 70 °C and maintained for 30 min. After cooling, the crude product was collected by filtration, poured into deionized water (200 mL) and adjusted the pH to 9 with ammonia water. The precipitate was washed by chloroform, dried under vacuum at 60 °C to obtain pure TAPP (1.2 g, 86%). ^1H NMR (500 MHz, DMSO-d_6) δ (ppm): 8.88 (s, 8H), 7.85 (d, J = 8.0, 8H), 6.99 (d, J= 8.0, 8H), 5.56 (s, 8H), -2.74(s, 2H).

5,10,15,20-Tetrakis-(4-aminophenyl)-porphyrin-Zn-(II) [1]

TAPP (200 mg, 0.3 mmol), $Zn(OAc)_2{\cdot}2H_2O$ (264 mg, 1.2 mmol) was added in a 250 ml three-neck round-bottom flask under nitrogen, a premixed solvent of methanol (20 mL), chloroform (90 mL) and DMF (30 mL) were added. The mixture was kept stirring for 24 h at 80 °C. After cooling to room temperature, the solution was transferred into a separatory funnel and washed with water (3 × 100 mL). The organic layer was combined, dried over Na_2SO_4 and concentrated in vacuo to afford TAPP-Zn as a dark green solid (190 mg, 87% yield). ^1H NMR (500 MHz, DMSO-d_6) δ (ppm): 8.83 (s, 8H), 7.79 (d, J = 8.0, 8H), 6.97 (d, J = 8.0, 8H), 5.45 (s, 8H).

Synthesis of Zn-Por-TT COF

14.8 mg Zn-TAPP (1 eq. 0.02 mmol) and 8 mg TT (2 eq. 0.04 mmol) was added in a 10 ml schlenk tube, subsequently, BnOH (250 μL) and DCB (750 μL) was added and sonicated for 15 min followed by addition of 6 M acetic acid (300 μL). The mixture was further

sonicated for another 2 minutes and degassed by three freeze–pump–thaw cycles, purged with N_2 and heated to 120 °C for 3 days. Upon cooling to r.t., the precipitate was collected by filtration and washed by DMAc, DCM, THF and methanol, dried under vacuum to afford a dark purple powder (16 mg, 75%). Elemental analysis (wt.%) calcd. for $\{C_{60}H_{36}N_8S_4Zn\}_n$: C 72.29, H 3.23, N 11.29, S 12.90; found: C 67.65, H 3.82, N 10.75, S 10.33.

Synthesis of Por-TT COF

13.5 mg TAPP (1 eq. 0.02 mmol) and 8 mg TT (2 eq. 0.04 mmol) was added in a 10 ml Schlenk tube, subsequently, BnOH (750 μL) and DCB (250 μL) was added and sonicated for 5 min followed by addition of 6 M acetic acid (300 μL). The mixture was further sonicated for another 2 minutes and degassed by three freeze–pump–thaw cycles, purged with N_2 and heated to 120 °C for 3 days. Upon cooling to r.t., the precipitate was collected by filtration and washed by DMAc, DCM, THF and methanol, dried under vacuum to afford a dark purple powder (14 mg, 70%). Elemental analysis (wt.%) calcd. for $\{C_{60}H_{38}N_8S_4\}_n$: C 72.14, H 3.80 , N 11.22, S 12.83; found: C 61.84, H 3.80, N 9.21, S 10.18.

Synthesis of COF-366

19.6 mg TAPP (1 eq. 0.03 mmol) and 8 mg BDT (2 eq. 0.06 mmol) was added in a 10 ml Schlenk tube, subsequently, EtOH (1 mL) and mesitylene (1 mL) was added and sonicated for 5 min followed by addition of 6 M acetic acid (200 μL). The mixture was further sonicated for another 2 minutes and degassed by three freeze–pump–thaw cycles, purged with N_2 and heated to 120 °C for 3 days. Upon cooling to r.t., the precipitate was collected by filtration and washed by DMAc, DCM, THF and methanol, dried under vacuum to afford a dark purple powder (18 mg, 70%).

Synthesis of COF-366-Zn

COF-366-Zn was synthesized according to the previously literature.[3] 100 mg (1 eq.) COF-366, 50 mg (2 eq.) $Zn(OAc)_2$ and EtOH (50 mL) were added into a 100 mL round-bottom flask, the mixture was allowed to stir at 40 °C for 12 h. The afford purple powder was adequately washed by deionized water, DMF, THF, acetone and dried under vacuum to obtain COF-366-Zn (98 mg, 93 %).

Section S3. SEM and TEM images

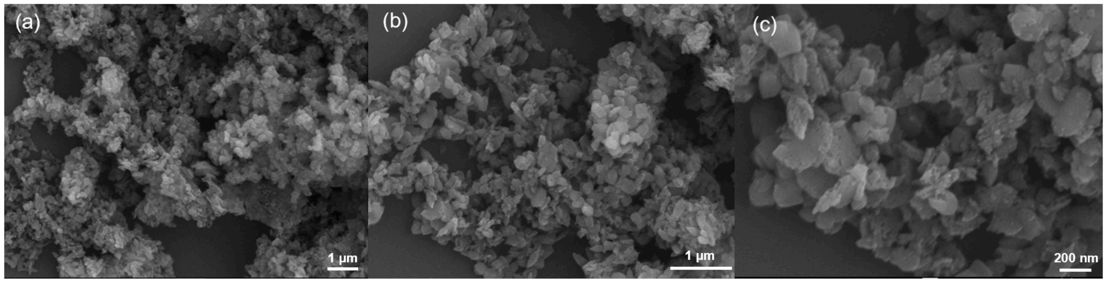

Figure S1. (a-c)SEM images of Zn-Por-TT COF

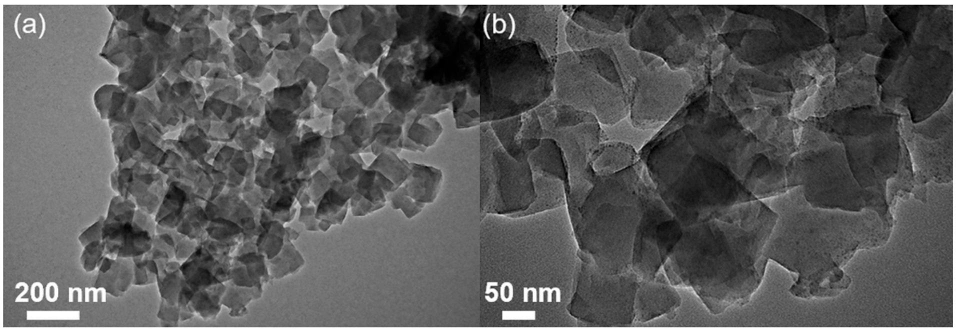

Figure S2. (a,b)TEM images of Zn-Por-TT COF

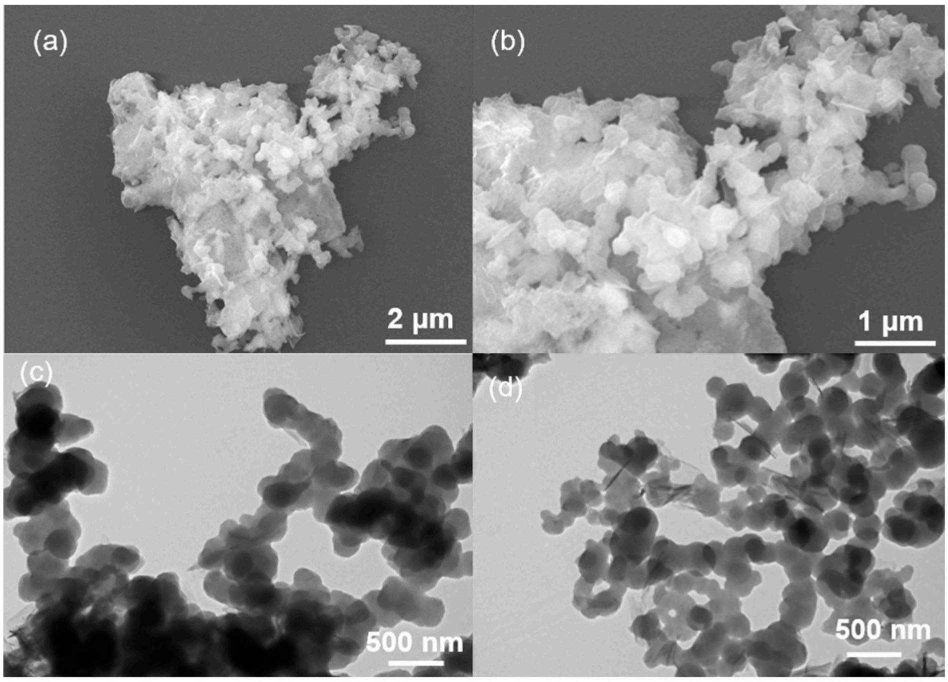

Figure S3. (a, b) SEM and (c, d) TEM images of COF-366-Zn

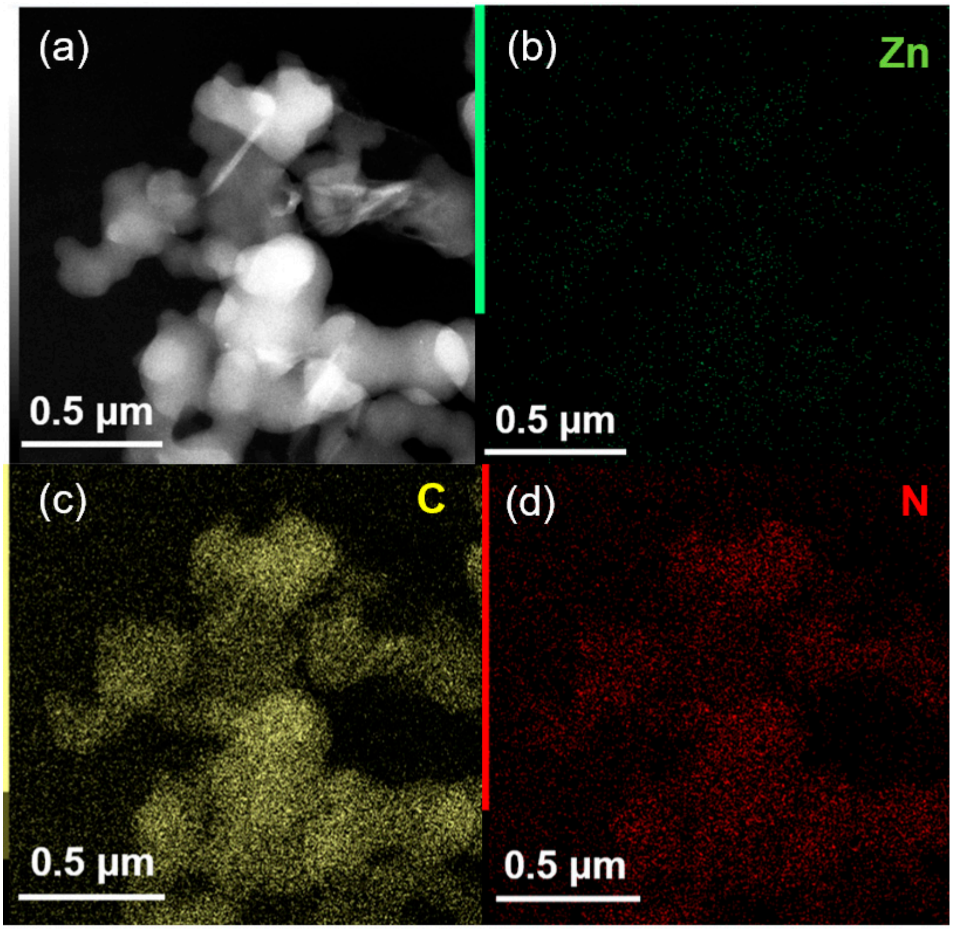

Figure S4. (a-d)Elemental maps of COF-366-Zn

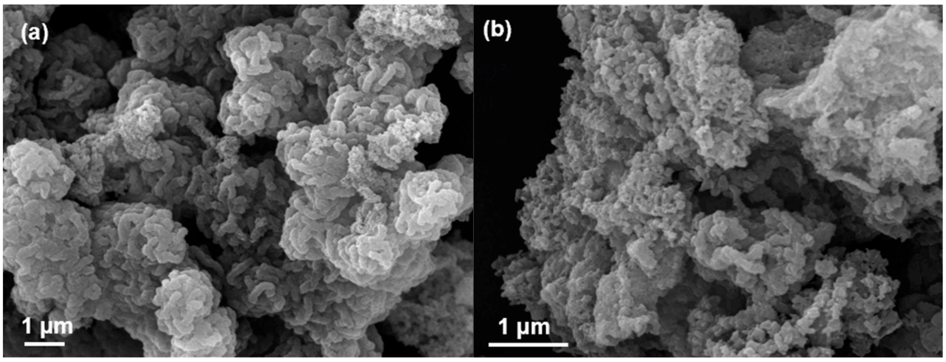

Figure S5. (a,b)SEM images of Por-TT COF

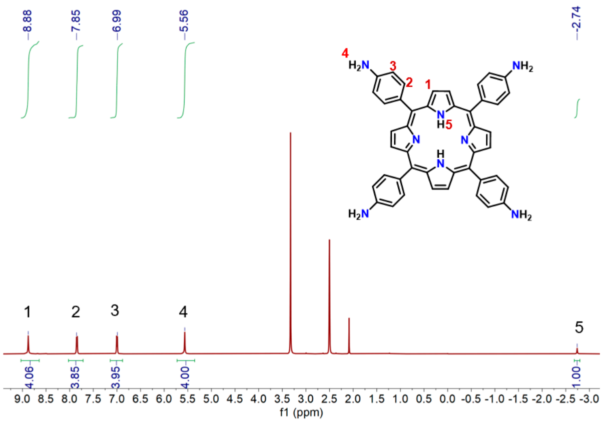

Figure S6. ^{1}H NMR spectrum of TAPP in DMSO-d_6

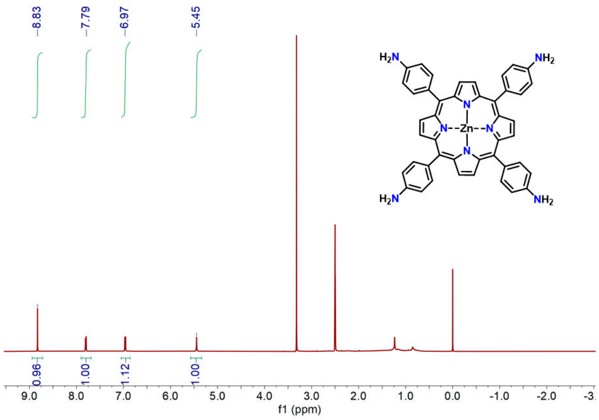

Figure S7. ^{1}H NMR spectrum of Zn-TAPP in DMSO-d_6

Table S1. Atomic coordinates of the AA-stacking mode of Zn-Por-TT COF using DFTB+ method

Space group: *P*1<br>*a* = 27.2545 Å, b = 27.3419 Å and *c* = 4.2925 Å.<br>*α* = 76.31°, *β* = 100.92° and *γ* = 95.77°			
	**X**	**Y**	**Z**
**C1**	0.37214	0.39465	0.55632
**C2**	0.40717	0.36303	0.54415
**C3**	0.454	0.39248	0.52246
**N4**	0.4471	0.44177	0.52512
**C5**	0.39749	0.44373	0.54619
**C6**	0.37376	0.48687	0.54974
**C7**	0.50042	0.68242	0.48061
**C8**	0.28851	0.5174	0.31301
**C9**	0.23745	0.51774	0.3106

**C10**	0.21438	0.48388	0.55197
**C11**	0.24468	0.44919	0.78876
**C12**	0.29582	0.44912	0.78945
**C13**	0.04174	0.53918	0.24738
**C14**	0.9968	0.52152	0.36733
**S15**	0.064	0.46077	0.7305
**C16**	0.08134	0.50966	0.42037
**C17**	0.13293	0.51485	0.37712
**N18**	0.16323	0.48256	0.5708
**C19**	0.62786	0.6054	0.44473
**C20**	0.59287	0.63692	0.45657
**C21**	0.54602	0.6075	0.47831
**N22**	0.55294	0.55822	0.47587
**C23**	0.60249	0.55628	0.45497
**C24**	0.62626	0.51309	0.4509
**C25**	0.49956	0.31759	0.51961
**C26**	0.71146	0.48257	0.68721
**C27**	0.76256	0.48221	0.68949
**C28**	0.78562	0.51608	0.44805
**C29**	0.75531	0.55081	0.21133
**C30**	0.70412	0.55087	0.21104
**C31**	0.95822	0.46074	0.75195
**C32**	0.00315	0.47836	0.63199
**S33**	0.93596	0.53914	0.26878
**C34**	0.91864	0.49026	0.57919
**C35**	0.86704	0.48511	0.6227
**N36**	0.83672	0.51737	0.42889
**C37**	0.58845	0.38321	0.4329
**C38**	0.62368	0.42253	0.4175
**C39**	0.60074	0.46567	0.45659
**N40**	0.55141	0.45249	0.48767
**C41**	0.54356	0.40217	0.48118
**C42**	0.49866	0.3724	0.50745
**C43**	0.31899	0.48348	0.55223
**C44**	0.4657	0.29461	0.29833
**C45**	0.46611	0.24341	0.30613
**C46**	0.50056	0.21253	0.53979
**C47**	0.53478	0.23565	0.76128
**C48**	0.53421	0.28683	0.75128
**C49**	0.4556	0.04608	0.26954
**C50**	0.47824	0.99928	0.37803
**S51**	0.53392	0.0593	0.71261
**C52**	0.48185	0.08224	0.42911
**C53**	0.47236	0.1345	0.38968

**N54**	0.50418	0.16085	0.56042
**C55**	0.41151	0.61684	0.56688
**C56**	0.37626	0.5775	0.58232
**C57**	0.39921	0.53435	0.54343
**N58**	0.44856	0.54749	0.51223
**C59**	0.45639	0.59789	0.51865
**C60**	0.5013	0.62761	0.49296
**C61**	0.68102	0.5165	0.44843
**C62**	0.53427	0.70542	0.70183
**C63**	0.5339	0.75662	0.69411
**C64**	0.49947	0.78748	0.46011
**C65**	0.46526	0.76441	0.23859
**C66**	0.46581	0.7132	0.24872
**C67**	0.54453	0.954	0.72962
**C68**	0.52189	0.00081	0.62097
**S69**	0.4662	0.94078	0.28657
**C70**	0.51825	0.91783	0.57011
**C71**	0.52773	0.86556	0.60991
**N72**	0.49585	0.8392	0.43917
**Zn73**	0.5	0.49999	0.5003
**H74**	0.33305	0.3841	0.57422
**H75**	0.40063	0.32301	0.5509
**H76**	0.30508	0.54393	0.11958
**H77**	0.2151	0.54472	0.11781
**H78**	0.22785	0.42255	0.9796
**H79**	0.31862	0.42253	0.9814
**H80**	0.0444	0.5714	0.04362
**H81**	0.14453	0.54655	0.18292
**H82**	0.66696	0.61589	0.42658
**H83**	0.5994	0.67701	0.44976
**H84**	0.69495	0.45602	0.88073
**H85**	0.7849	0.45521	0.88202
**H86**	0.7721	0.57743	0.02053
**H87**	0.68136	0.57749	0.0193
**H88**	0.95557	0.4285	0.95592
**H89**	0.85548	0.45346	0.81736
**H90**	0.59337	0.34546	0.40606
**H91**	0.66161	0.42169	0.37391
**H92**	0.43925	0.31757	0.10798
**H93**	0.43969	0.22729	0.12395
**H94**	0.56191	0.21264	0.94577
**H95**	0.56107	0.30365	0.92745
**H96**	0.42208	0.05255	0.08284
**H97**	0.43839	0.14983	0.21748

**H98**	0.40661	0.65457	0.59369
**H99**	0.33837	0.57835	0.62589
**H100**	0.56073	0.68243	0.89251
**H101**	0.5603	0.77273	0.87626
**H102**	0.43818	0.7874	0.05388
**H103**	0.43895	0.69637	0.0725
**H104**	0.57803	0.94751	0.91638
**H105**	0.56166	0.85022	0.78215

Reference

[1] Lu, M.; Liu, J.; Li, Q.; Zhang, M.; Liu, M.; Wang, J.; Yuan, D.; Lan, Y. Rational Design of Crystalline Covalent Organic Frameworks for Efficient CO_2 Photoreduction with H_2O. *Angew. Chem. Int. Ed*. **2019**, 58, 12392.

[2] Zhu, J.; Liu, E.; Gao, Y.; Yang, W.; Huang, C.; Liu, F. A Conjugated Copper Porphyrin Polymer for Catalytic Coupling of Acetonitrile and Alcohols. *ChemistrySelect.* **2019**, *4*, 10653.

[3] Liu, M.; Chen, Y.; Huang, X.; Dong, L.; Lu, M.; Guo, C.; Yuan, D.; Chen, Y.; Xu, G.; Li, S.; Lan, Y. Porphyrin-Based COF 2D Materials: Variable Modification of Sensing Performances by Post-Metallization. *Angew. Chem. Int. Ed*. **2022**, *61*, e202115308.
